# Geographical Variation of COPD Mortality and Related Risk Factors in Jiading District, Shanghai

**DOI:** 10.3389/fpubh.2021.627312

**Published:** 2021-02-03

**Authors:** Qian Peng, Na Zhang, Hongjie Yu, Yueqin Shao, Ying Ji, Yaqing Jin, Peisong Zhong, Yiying Zhang, Honglin Jiang, Chunlin Li, Ying Shi, Yingyan Zheng, Ying Xiong, Zhengzhong Wang, Feng Jiang, Yue Chen, Qingwu Jiang, Yibiao Zhou

**Affiliations:** ^1^Jiading District Center for Disease Control and Prevention, Shanghai, China; ^2^Fudan University School of Public Health, Shanghai, China; ^3^Key Laboratory of Public Health Safety, Fudan University, Ministry of Education, Shanghai, China; ^4^Fudan University Center for Tropical Disease Research, Shanghai, China; ^5^Faculty of Medicine, School of Epidemiology and Public Health, University of Ottawa, Ottawa, ON, Canada

**Keywords:** COPD, traffic-related pollutant, temperature, GWLR, mortality

## Abstract

**Background:** Chronic obstructive pulmonary disease (COPD) is the fourth leading cause of death in China. Although numerous studies have been conducted to determine the risk factors for COPD mortality such as ambient air pollution, the results are not fully consistent.

**Methods:** This study included mortality analysis and a case-control design by using the data extracted from the Mortality Registration System in Jiading District, Shanghai. Traditional logistic regression, geographically weighted logistic regression (GWLR), and spatial scan statistical analysis were performed to explore the geographic variation of COPD mortality and the possible influencing factors.

**Results:** Traditional logistic regression showed that extreme lower temperature in the month prior to death, shorter distance to highway, lower GDP level were associated with increased COPD mortality. GWRL model further demonstrated obvious geographical discrepancies for the above associations. We additionally identified a significant cluster of low COPD mortality (OR = 0.36, *P* = 0.002) in the southwest region of Jiading District with a radius of 3.55 km by using the Bernoulli model. The geographical variation in age-standardized mortality rate for COPD in Jiading District was explained to a certain degree by these factors.

**Conclusion:** The risk of COPD mortality in Jiading District showed obvious geographical variation, which were partially explained by the geographical variations in effects of the extreme low temperature in the month prior to death, residential proximity to highway, and GDP level.

## Introduction

The 2017 Global Burden of Science Study (GBD) found that the total number of deaths due to chronic respiratory diseases increased by 18.0%, from 3.32 (95% CI: 3.01 to 3.43) million in 1990 to 3.91 (95% CI: 3.79 to 4.04) million in 2017 (1). Chronic obstructive pulmonary disease (COPD), the most common type of chronic respiratory disease, is characterized by airflow obstruction that can progress to pulmonary heart disease and respiratory failure. The China Pulmonary Health (CPH) Study, which was carried out among 50,991 adults aged 20 years or older during 2012-2015, reported an overall prevalence of COPD being 8.6% (11.9% in men and 5.4% in women) (2). COPD is currently the fourth leading cause of death in China (3), making it a one of most pressing health issues in the country.

Smoking is a major cause of COPD (4) and ambient air pollutants play an important part in COPD exacerbation (5, 6), and both are related to increased mortality and decreased quality of life. Previous studies have shown high heterogeneity in geographic variation in COPD mortality, which may be associated with personal characteristics, air pollution and other factors such as temperature (7) or may be due to study limitations. Many studies used ecological time-series method to determine the effect of air pollutants on COPD (7) and low spatiotemporal resolution of measurements of ambient air pollution obtained from fixed monitoring stations as a surrogate for individual exposure, and ecological fallacy is a concern. A study found traffic-related pollution was affected by many factors including dynamic emissions, measurement distance and height from the roadway, street canyon, obstructions along the way, and meteorological conditions such as wind direction and speed (8). When the exposure concentration was highly spatially and geographically heterogeneous, mis-estimation of exposures would be introduced into analysis (9). In recent years, multi-pollutant indicators have been adopted to investigate the association with health outcomes to better evaluate real-world exposure to pollutants such as residential distance to the closest roads (10), factory density (11), and traffic density (12). Residential proximity to road has been found to be a good proxy for traffic-related air pollutant concentrations (13).

The current study aimed to determine the geographical variation in COPD mortality risk, and to explore the geographical variations in the effects of ambient air pollution indicators and other factors on COPD mortality. We used geographically weighted regression model (GWR) to explore the spatial variation in the risk of COPD death and relevant driving factors at a local level. GWR is an extension of weighted regression by establishing the local regression equation at each point in the space scope, where the weights are defined by the geographical distance between objectives (14), and has been to study different diseases (15, 16).

## Materials and Methods

### Study Site

This study was conducted in Jiading District, located in the northwest of Shanghai. The district covers an area of 464.2 km^2^ and 12 towns with a registered population of 658,200. Jiading District is situated in the northern edge of northern subtropical region with abundant precipitation, warm and humid climate, and adequate sunshine. The annual mean temperature was 17.6°C in 2018, while the lowest temperature reached −6.4°C in the same year (17). Since 2010, the roads system in Jiading District has been gradually developing into a network, with a total length of 1,407 km. Meanwhile, the main traffic network of the district currently includes five vertical and six horizontal lines.

### Study Design

We performed a population-based case-control study by using the data extracted from the Mortality Registration System in Jiading District, Shanghai. The underlying causes of death coded by the International Statistical Classification of Diseases, 10th revision (ICD-10), were reclassified following the method of GBD study (3). Cases were the deaths due to COPD between 2011 and 2018 with complete information on age, sex, education, occupation, marriage, date of death, residential address. Two groups of controls were selected from deaths due to cerebrovascular disease and due to tumor. Frequency matching was used to match the two control groups to the case group with age (5 years or less), sex and year of death. The study included a total of 3,438 deaths (1,146 COPD cases, 1,146 cerebrovascular controls, and 1,146 tumor controls).

### Data Collection

#### Population Data

Population data from 2011 to 2018 were taken from the Public Security Bureau in Jiading District. Age-standarized mortality rates (ASMR) were calculated according to sex, town, and 5-year time period.

#### Vector Data

We collected the following vector data. The map of Jiading District and roads data in 2010 were purchased from the Shanghai Digital Bitmap Information Technology Co., LTD. The roads in Jiading District were divided into main roads (national, provincial, and some county roads with a traffic volume of 10,000 counts/day) and highways (Chinese Expressways). The geographical addresses of factories and public hospitals were obtained from the Jiading District Center for Disease Control and Prevention (CDC), and then we screened out factories that produce air pollutants according to the detection report of factory and technological process. Finally, the geographical addresses of factories and public hospitals were converted into latitude and longitude by using Geocoding software (https://www.esri.com/en-us/arcgis/products/geocoding) and then transformed into vector point data in ArcMap 10.5 (Environmental Systems Research Institute, Inc, Redlands, CA).

#### Raster Data

Gross domestic product (GDP), population density, and extreme low temperature in the month prior to death were stored as raster data. China's GDP and population per kilometer grid in 2010 were downloaded from the Global Change Research Data Publishing & Repository, with a spatial resolution of 1 km. Previous studies have found that extreme low temperature can cause more serious disease burden of COPD than high temperature (18), and the cold effect can be delayed for up to a month (19). Therefore, the average extreme low temperature in the month prior to death was used to explore the impact of temperature on COPD death. The extreme low temperature in China by day from December 1, 2010 to November 31, 2018 was downloaded from the Chinese Meteorological Data Network (http://data.cma.cn). Meanwhile, raster data of GDP, population and temperature in Jiading District were extracted by the mask extraction tool in ArcMap10.5.

#### Data Processing

Firstly, we used the nearest neighbor analysis tool in ArcMap10.5 to calculate distance. The distance between residential address and the closest road was calculated to evaluate individual traffic-related air pollution, the distance to the closest factory was used to represent individual industrial-related air pollution, and the distance to the closest public hospital was used as an indicator of individual health care accessibility. Then, we also calculated the factory density according to geographical distribution of factories, in which the deceased once lived, by using a kernel density analysis tool in ArcMap 10.5. Finally, we superimposed the residential address with raster data and evaluated individual exposure level by using the Extract Values to Points tool in ArcMap 10.5. In this study, GDP was used as an indicator of the economic status of the dead and population per kilometer was adopted to describe population distribution in Jiading District. Besides, temperature represented the average extreme low temperature in the month prior to death in this study.

#### Exposure Classification

We classified socioeconomic factors including marriage (married or others), education (senior high school or above, junior middle school, primary school or below) and occupation (industry-related, agriculture-related, others). Extreme low temperature in the month prior to death was classified into four levels (<5, 5–15, 16–20, >20°C) with >20°C as the reference group. We classified the following distances from the residential address to the closest highways (<200, 200–350, 351–650, >650 m), to the closest major road (<100, 100–300, 301–500, >500 m), to the closest public hospital (<200, 200–500, 501–1,000, 1,001–2,000, >2,000), and to the closest factory (<50, 50–200, 201–500,>500 m), with the most distant group as the reference group for each. We classified residential factory density, GDP, and population density into quartiles (Q1, Q2, Q3, Q4), with Q4 served as the reference group.

### Analytic Methods

#### Age-Standardized Mortality

The average ASMR for COPD between 2011 and 2018 was calculated using the Chinese population in 2010 as standard population, and visualized in ArcMap10.5.

#### Non-spatial Logistic Regression

For the case-control study, we firstly used simple logistical regression to analyze the association between COPD and each risk factor by using SPSS (SPSS Inc., USA), and significant factors were then included in multivariable logistical regression. Considering the seasonality in COPD mortality, we also divided the death data into cold season (from September to February) and warm season (from March to August), and then we conducted non-spatial logistical regressions in the same method as above-mentioned, respectively.

#### Geographically Weighted Logistic Regression (GWLR)

A GWLR model was used to analyze the associations between COPD mortality and all the risk factors using the data from the case-control study. The relationship may be described as:

$$\begin{array}{l} \log\left(\frac{P\left(y_{i}=1\right)}{1-P\left(y_{i}=1\right)}\right)=\beta_{0}\left(u_{i},v_{i}\right)+\overset{k}{\underset{j=1}{\sum }}\beta_{j}\left(u_{i},v_{i}\right)x_{ij} \end{array}$$

where y_i_ and x_ij_, denote the cause of death and explanatory variables for individual i with location coordinates (u_i_, v_i_), and β_0_ (u_i_, v_i_) and β_j_ (u_i_, v_i_) are the location-specific intercept and coefficients.

In this GWLR model, we combined two control groups. Adaptive bi-square was used as the kernel type, golden section search as the bandwidth selection method and AICc as criteria. Variables included in the model were extreme low temperature in the month prior to death, distance to the closest highway, and GDP. Data analysis was conducted by using GWR4.0 software. ArcMap10.5 software was used to map the results of the GWLR model.

#### Spatial Scan Statistical Analysis

The spatial scan statistical analysis was widely applied to detect clustering of cases by using cylindrical scanning windows with dynamic changes in size and position (20). The expected and observed number of cases were used to generate the log likelihood ratio (LLR). Then, the scanning window with the largest LLR value was selected as the high-clustering window. The spatial and temporal information involved in the window was collected and determined. Finally, the statistical significance of those clusters was tested by using the Monte Carlo testing method. In this study, data from 1,146 COPD cases and 2,292 non-COPD controls fit the Bernoulli model with a 6-month interval. The analysis was carried out by SaTScan9.6 software (https://www.satscan.org/) and then visualized the significant spatial cluster in ArcMap 10.5.

## Results

### The Average ASMR

**Figure 1:**
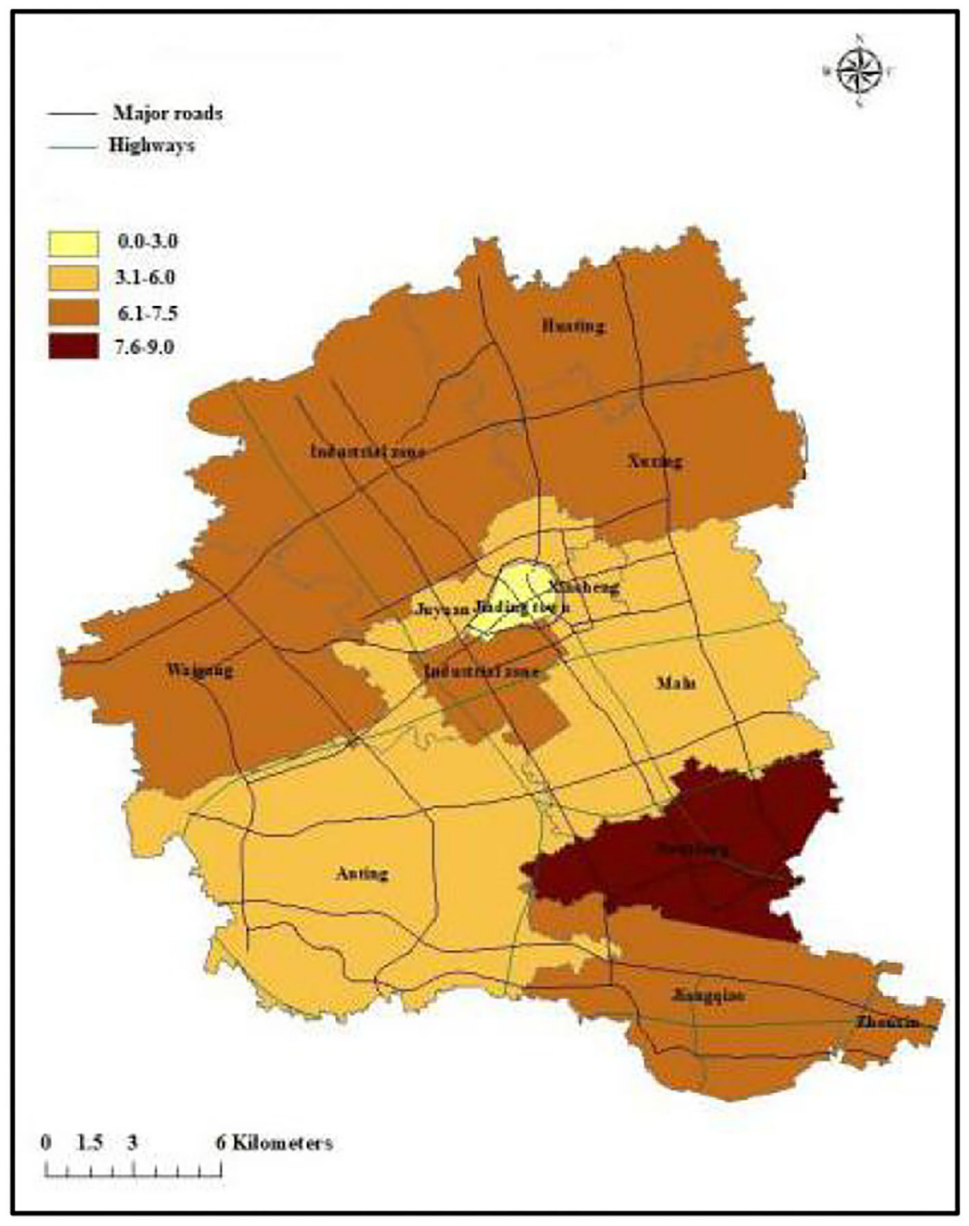
The map of ASMR caused by COPD in Jiading District, Shanghai during 2011 and 2018.

Figure 1 showed that the average ASMR due to COPD was the lowest in Jiading town. We observed a lower risk of COPD mortality from the northeast corner of Jiading District across the southwest corner of this district. Besides, the ASMR in the north, northwest, and southeast of Jiading District appeared to be higher than other areas.

### Non-spatial Logistic Regression

Case and control groups are frequently matched by sex (male: 69.1%, female: 30.9% and age (83.8 ± 7.7 for COPD deaths, 83.4 ± 7.4 for cerebrovascular disease deaths, and 82.8 ± 7.1 for tumor deaths, respectively), and there were no significant differences in sex and age distributions between cases and controls.

Table 1 showed the results from logistic regression models based on data from 1,146 COPD cases and 1,146 cerebrovascular controls. In the adjusted model, industry- related occupation (vs. others, aOR = 1.414, 95% CI: 1.079–1.853), the extreme lower temperature (<5°C vs. >20°C, aOR = 1.496, 95% CI: 1.169–1.915), shorter distance to the closest highway (<200 m vs. >650 m, aOR = 1.819, 95% CI: 1.134–2.919), and lower GDP level (Q1 vs. Q4, aOR = 1.505, 95% CI: 1.019–2.225) were significantly associated with increased risk of COPD death. In addition, population density Q3 was significantly and negatively associated with the risk of COPD death (aOR = 0.449, 95% CI: 0.322–0.627) compared to Q4.

**Table 1:** The association of COPD mortality and risk factors from non-spatial logistical regressions using cerebrovascular disease deaths as controls.

**Variables**	**Univariate analysis**		**Multivariate analysis**	
	**OR (95%CI)**	***P***	**aOR (95%CI)**	***P***
**Education**				
Senior high school or above	1			
Junior middle school	1.478 (0.875–2.498)	0.144		
Primary School or below	1.716 (1.035–2.845)	0.036^*^		
**Marriage**				
Others	1			
Married	0.954 (0.809–1.124)	0.571		
**Occupation**				
Others	1		1	
Industry- related	1.455 (1.131–1.872)	0.004^*^	1.414 (1.079–1.853)	0.012^*^
Agriculture-related	1.441 (1.199–1.734)	<0.001^*^	1.157 (0.921–1.453)	0.210
**Extreme low temperature (°C)**				
>20	1		1	
15–20	1.085 (0.810–1.454)	0.584	1.111 (0.796–1.552)	0.535
5–15	1.245 (0.993–1.561)	0.057	1.163 (0.900–1.503)	0.250
<5	1.569 (1.262–1.950)	<0.001^*^	1.496 (1.169–1.915)	0.001^*^
**Distance to the closest highway (m)**				
>650	1		1	
350–650	1.323 (1.027–1.704)	0.030^*^	1.509 (1.131–2.013)	0.005^*^
200–350	1.473 (1.114–1.949)	0.007^*^	1.710 (1.243–2.354)	0.001^*^
<200	1.691 (1.118–2.560)	0.013^*^	1.819 (1.134–2.919)	0.013^*^
**Distance to the closest major road (m)**				
>500	1			
300–500	0.867 (0.690–1.088)	0.218		
100–300	0.786 (0.637–0.969)	0.024^*^		
<100	0.660 (0.519–0.841)	0.001^*^		
**Distance to the closest hospital (m)**				
>2,000	1		1	
1,000–2,000	1.164 (0.933–1.453)	0.177	1.388 (1.065–1.808)	0.015^*^
500–1,000	0.813 (0.654–1.010)	0.061	1.147 (0.862–1.526)	0.348
200–500	0.736 (0.569–0.953)	0.020^*^	1.307 (0.943–1.812)	0.108
<200	0.509 (0.309–0.840)	0.008^*^	0.910 (0.518–1.599)	0.744
**Distance to the closest factory (m)**				
>500	1			
200–500	0.906 (0.701–1.170)	0.448		
50–200	0.927 (0.719–1.197)	0.563		
<50	1.093 (0.850–1.405)	0.490		
**Factory density**				
Q4	1			
Q3	1.254 (0.995–1.581)	0.055		
Q2	1.018 (0.808–1.283)	0.879		
Q1	1.208 (0.958–1.523)	0.111		
**Population density**				
Q4	1		1	
Q3	0.520 (0.392–0.691)	<0.001^*^	0.449 (0.322–0.627)	<0.001^*^
Q2	0.893 (0.661–1.206)	0.461	0.625 (0.414–0.945)	0.026^*^
Q1	1.058 (0.785–1.427)	0.711	0.932 (0.623–1.392)	0.729
**GDP**				
Q4	1		1	
Q3	1.028 (0.813–1.300)	0.816	0.764 (0.572–1.020)	0.068
Q2	1.109 (0.881–1.396)	0.379	0.831 (0.602–1.148)	0.262
Q1	1.994 (1.575–2.524)	<0.001^*^	1.505 (1.019–2.225)	0.040^*^

*Temperature represented the average extreme low temperature in the month prior to death*.

*Q1–Q4 represent the interquartile value of risk factors*.

*OR, Odds ratio; aOR, adjusted odds ratio; CI, confidence interval*.

* *P < 0.05*.

When we used tumor deaths as controls, the results were similar (Table 2). In the multivariable logistic regression model, the extreme lower temperature (<5°C vs. >20°C, aOR = 2.052, 95% CI: 1.611–2.613), shorter distance to the closest highway (200–350 m vs. >650 m, aOR = 2.119, 95% CI: 1.521–2.951), shorter distance to the closest factory (200–500 m vs. >500 m, aOR = 1.595, 95% CI: 1.218–2.090), and lower GDP level (Q1 vs. Q4, aOR = 1.795, 95% CI: 1.179–2.733) were significantly associated with increased risk of COPD death. Besides, married people (aOR = 0.754, 95% CI: 0.625–0.909) showed lower risk of COPD death (Table 2). Furthermore, although we divided the deaths into cold season (from September to February) and warm season (from March to August), we still found that extreme lower temperature, shorter distance to the closest highway, lower GDP level were associated with higher risk of COPD mortality (Supplementary Table 1).

**Table 2:** The association of COPD mortality and risk factors from non-spatial logistical regressions using tumor deaths as controls.

**Variables**	**Univariate analysis**		**Multivariate analysis**	
	**OR (95%CI)**	***P***	**OR (95%CI)**	***P***
**Education**				
Senior high school or above	1			
Junior middle school	1.861 (1.115–3.104)	0.017^*^		
Primary School or below	2.325 (1.419–3.808)	0.001^*^		
**Marriage**				
Others	1		1	
Married	0.730 (0.619–0.862)	<0.001^*^	0.754 (0.625–0.909)	0.003^*^
**Occupation**				
Others	1			
Industry- related	1.295 (1.011–1.658)	0.041^*^		
Agriculture-related	1.363 (1.136–1.636)	0.001^*^		
**Extremely low temperature (°C)**				
>20	1		1	
15–20	1.039 (0.782–1.380)	0.793	1.032 (0.752–1.415)	0.846
5–15	1.563 (1.248–1.957)	<0.001^*^	1.598 (1.239–2.056)	<0.001^*^
<5	2.174 (1.750–2.701)	<0.001^*^	2.052 (1.611–2.613)	<0.001^*^
**Distance to the closest highway (m)**				
>650	1		1	
350–650	1.091 (0.856–1.391)	0.482	1.390 (1.045–1.848)	0.024^*^
200–350	1.702 (1.272–2.277)	<0.001^*^	2.119 (1.521–2.951)	<0.001^*^
<200	1.148 (0.785–1.678)	0.477	1.180 (0.761–1.829)	0.460
**Distance to the closest major road (m)**				
>500	1			
300–500	0.807 (0.644–1.011)	0.062		
100–300	0.753 (0.610–0.929)	0.008^*^		
<100	0.699 (0.547–0.892)	0.004^*^		
**Distance to the closest hospital (m)**				
>2,000	1			
1,000–2,000	0.878 (0.704–1.094)	0.246		
500–1,000	0.708 (0.567–0.883)	0.002^*^		
200–500	0.587 (0.453–0.762)	<0.001^*^		
<200	0.471 (0.283–0.782)	0.004^*^		
**Distance to the closest factory (m)**				
>500	1		1	
200–500	0.819 (0.632–1.062)	0.132	1.595 (1.218–2.090)	0.001^*^
50–200	0.865 (0.667–1.122)	0.274	0.977 (0.744–1.283)	0.866
<50	0.979 (0.759–1.263)	0.871	1.349 (1.013–1.795)	0.040^*^
**Factory density**				
Q4	1			
Q3	1.568 (1.241–1.980)	<0.001^*^		
Q2	1.013 (0.803–1.278)	0.912		
Q1	1.257 (0.996–1.586)	0.054		
**Population density**				
Q4	1			
Q3	0.636 (0.482–0.839)	0.001^*^		
Q2	0.991 (0.739–1.328)	0.949		
Q1	1.126 (0.839–1.510)	0.428		
**GDP**				
Q4	1		1	
Q3	0.902 (0.714–1.138)	0.382	1.085 (0.796–1.479)	0.608
Q2	1.092 (0.865–1.378)	0.459	1.179 (0.816–1.703)	0.381
Q1	1.688 (1.335–2.133)	<0.001^*^	1.795 (1.179–2.733)	0.006^*^

*Temperature represented the average extreme low temperature in the month prior to death*.

*Q1–Q4 represent the interquartile value of risk factors*.

*OR, Odds ratio; aOR, adjusted odds ratio; CI, confidence interval*.

* *P < 0.05*.

### GWLR Model

**Figure 2:**
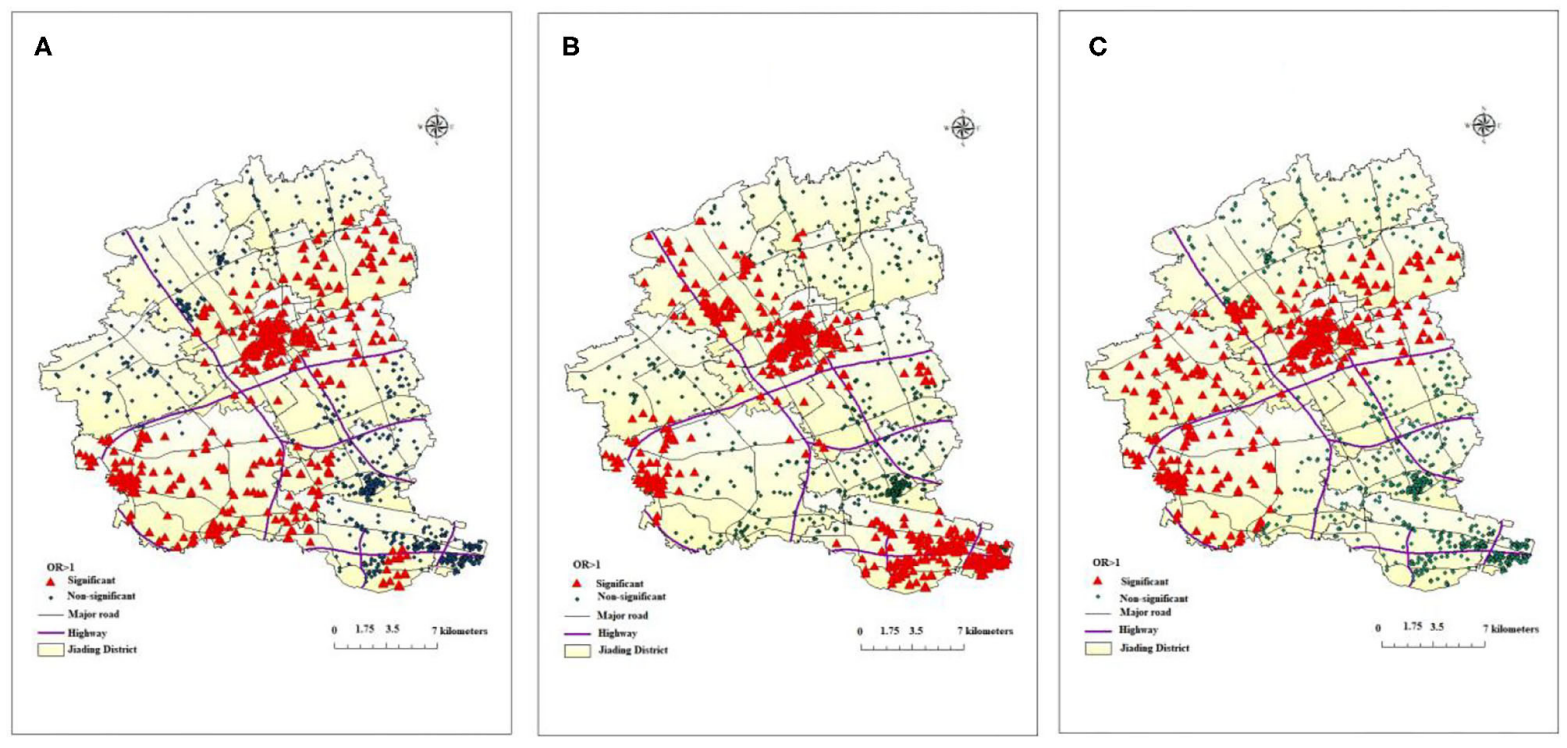
Geographical distribution of risk factors associated with COPD death from GWLR model: **(A)** (temperature), **(B)** (distance to the closest highway), **(C)** (GDP).

In this study, the best bandwidth size was 654. The value of AIC_C_ in the global regression was 3172.3, while the value was 2927.6 for GWLR, which indicated that the GWLR model fitted the data better than the regular regression model. Geographical distribution of risk factors associated with COPD mortality from GWLR model was presented in Figure 2. The OR values from GWR indicated that extreme lower temperature, shorter distance to highway and lower GDP increased the risk of COPD death, which were consistent with results from non-spatial logistic regression. However, GWR added that the spatial distribution of significant clusters for each risk factors were obviously different. Extreme low temperature (per 1°C reduce) increased COPD mortality (*P* < 0.05) for 40.2% of study individuals and the significant cluster areas marked in red (Figure 2A). Residential proximity to highway (per 1 km reduce) was positively (*P* < 0.05) associated with COPD mortality for 44.2% individuals (Figure 2B). GDP (per 1 unit reduce) also increased COPD mortality for 35.2% study individuals, marked in red (Figure 2C).

### Spatial Scan Statistical Analysis

**Figure 3:**
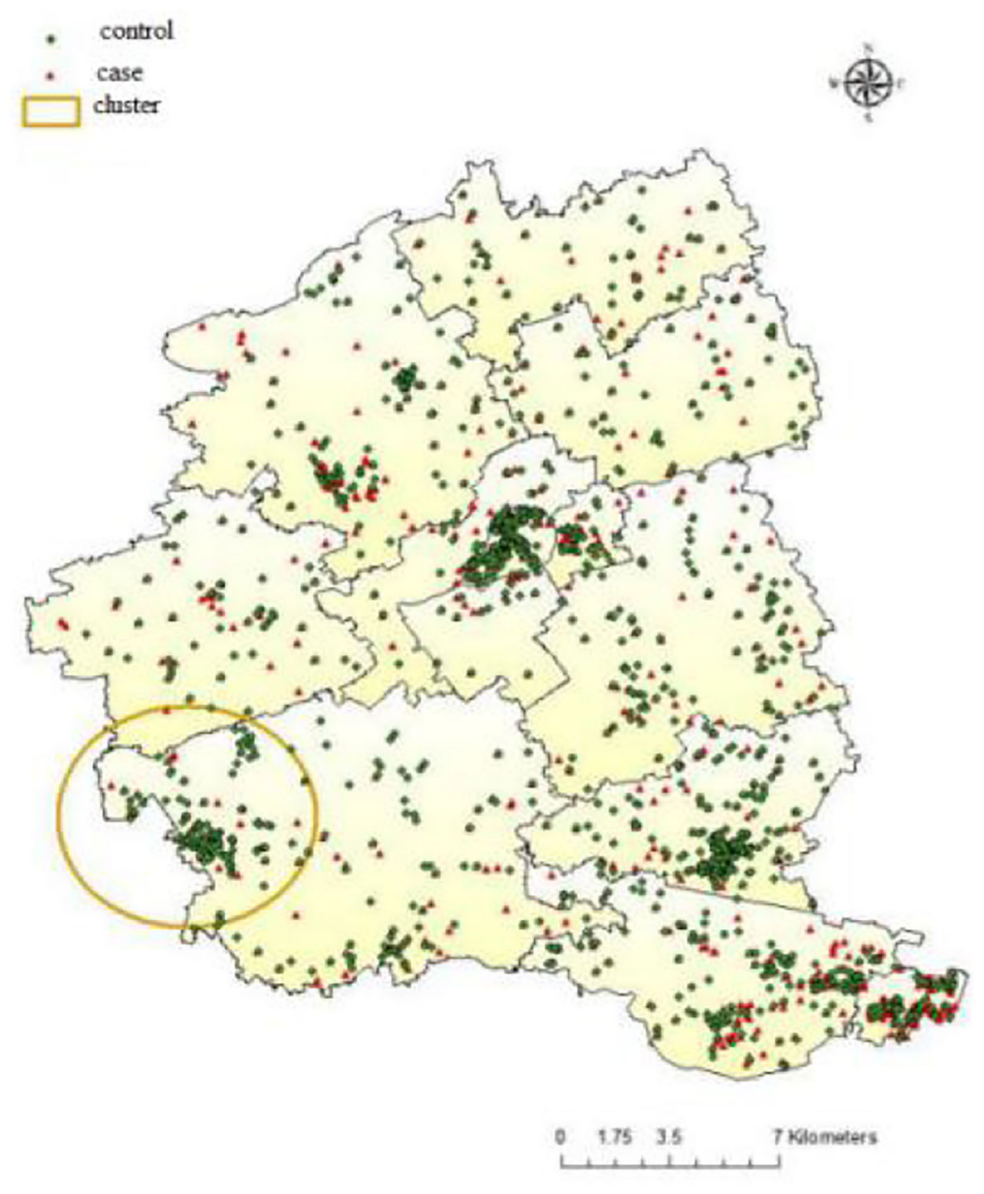
Distribution of COPD case and controls in Jiading District with the location of significant spatial cluster, 2011–2018.

Figure 3 presented the results of spatial scan statistical analysis by using Bernoulli model with 6-month interval, the triangles in red denoted the residential location of COPD cases while points in green denoted the residential location of controls. There was a significant cluster of lower risk of COPD mortality (OR = 0.36, *P* = 0.002) in the southwest of Jiading District, the cluster spanned the period from 2011/1/1 to 2014/12/31 with a radius of 3.55 km.

## Discussion

According to the geographic distribution of ASMR in Jiading district, Shanghai, lower ASMR appeared from the northeast to the southwest of Jiading District, while higher ASMR distributed in the north, northwest, and southeast of Jiading District, which highlighted the geographical variation of COPD mortality in Jiading District. The results from non-spatial logistic regression indicated extreme lower temperature, shorter distance to highway, lower GDP level might be associated with increased risk of COPD mortality. Besides, the results from GWLR model not only further verified the association between COPD mortality and the above risk factors but also added that the spatial distribution of significant clusters for each risk factors were obviously different. Finally, the Bernoulli model showed a significant cluster of reduced COPD mortality, which was consistent with the map of ASMR and could be interpreted as few traffic routes (according to road distribution in Jiading District), relatively higher temperature and higher GDP level in this area.

Previous study conducted in Ningbo found a reverse J-shape association between COPD mortality and average air temperature, both extreme low temperature (RR = 2.767, 95% CI: 1.950–3.928) and high temperature (RR = 1.197, 95% CI: 1.021–1.404) over lags of 0–14 days could increase COPD mortality (21). Another study also revealed that low temperature caused more serious effect on COPD mortality than high temperature by using the distributed lag non-linear model (18). A time-series panel study was carried out by measuring peak expiratory flow (PEF) and forced expiratory volume in one second (FEV_1_) in 28 male COPD patients repeatedly to estimate the cumulative effect of temperature on pulmonary function, and the results showed robust evidence that both low and high temperatures were significantly related to the decline of pulmonary function, PEF in particular (22).

Traffic becomes one of the major contributors to ambient air pollution in urban environments (23). A growing number of studies have used traffic as a measure of air pollution exposure to evaluate its negative effects on lung cancer (9), childhood asthma (24), and cardiovascular diseases (25). In this study, residential proximity to highways was a risk factor for COPD mortality while residential proximity to roads showed no significant association with COPD mortality. Previous studies showed consistent results for the relationship between COPD mortality and traffic-related pollution. A cross-sectional study conducted in Jiading District from 2001 to 2010 found that arterial road density was significantly associated with the mortality of COPD (26). Proximity to roads (0–200 m) and road density are predictors for COPD morbidity in Kandy (27). A cohort study performed by Cakmak et al. in Canada found that exposure to higher road density and proximity to major traffic roads were related to increased COPD mortality (28). However, they selected the length of local roads within a 200 m radius of postal code centroid rather than residential distance to the closest road as indicator, which might explain part of the geographical heterogeneity observed since different traffic parameters led to various sensitivity in estimates of exposure (11). The discrepancy between the results of residential proximity to highway and road might be due to a wide emission range of pollutants on expressways and a declined concentration with increasing distance (29).

In this study, we found people engaged in agriculture or industrial-related occupations, unmarried, and with lower education level showed higher risk of COPD death, which were consistent with previous studies. A national cross-sectional study of COPD showed that middle or high school education (OR = 0.76, 95% CI: 0.64–0.90) and college or higher education (OR = 0.47, 95% CI: 0.33–0.66) had lower risk of COPD (7). Previous studies have found that exposure to pollutants in the occupational environment would exacerbate airflow limitations in COPD patients and increase the risk of COPD death (30, 31). In addition, we used GDP as an indicator for social-economic status, and the results showed that lower GDP level was risk factor for COPD mortality.

The results in GWLR model further verified the relationship between COPD mortality and extreme low temperature in the month prior to death, residential proximity to highway, and GDP. We also found significant geographical variations in the effects of these factors. For example, the significant clusters of residential proximity to highway mainly located from the southeast across the northwest of Jiading District, adjacent to the highway. COPD mortality in northeast of Jiading District was less affected. In addition, a previous study showed that wind direction greatly affected the concentration of ambient pollutants (32). Due to the influence of southeast monsoon, the dominant wind direction of Jiading District is from the southeast in summer but northwest in winter, which could partially explain the distributions of the clusters. In addition, we selected a representative map of extreme low temperature in January 1, 2011 to describe the distribution of temperature in Jiading District. Relatively lower temperature and lower GDP might partially explain the higher ASMR in northern and northwest of Jiading District. Because of urban heat island effect (33), rapid social-economic development, and the policy that avoid building highways in downtown area, the center of Jiading District (Jiading town) had relatively higher temperature, higher GDP level, and longer distance to highway, which might attributed to the lowest ASMR of COPD. The Bernoulli model showed a significant cluster of reduced COPD mortality, which partially overlapped with the significant clusters of the extreme low temperature in the month prior to death, proximity to highway and GDP in the GWLR model, which indicated few traffic routes (according to road distribution in Jiading District), relatively higher temperature and higher GDP level in this area may explain the reduced COPD mortality cluster.

Our study had several strengths and limitations. In this study, conventional data and various land use variables at fine spatial resolutions were combined to explore the factors associated with COPD death and spatial distribution difference in a relatively novel method. Besides, comprehensive indicators were used to better reflect individual exposure to pollutants such as distance to the closest highway, factory density. However, our study also had several limitations, Firstly, we used China's GDP and population per kilometer grids in 2010 to represent the social-economic status and population density of the whole research period, and used roads data in 2010 to evaluate traffic-related pollution, which would lead to inaccurate assessment of present exposure. Secondly, due to the lack of individual information collection, the potential risk factors included in our study were incomplete, particularly the lack of data on smoking (34) and body mass index (BMI) (35), which might confound the associations in the current study. However, a study conducted in China found 2 years after being diagnosed, patients with COPD had smoking cessation rates of 62.8% (36). In addition, smoking is a predominant behavior for men, frequency matching by sex might reduce the confounding effects of smoking.

## Conclusion

The risk of COPD mortality in Jiading District showed an obvious geographical variation, which might partially be explained by geographical variations in the effects of the extreme low temperature in the month prior to death, residential proximity to highway, and GDP level. These factors should be taken into consideration when targeted interventions are developed at the local level to reduce COPD mortality risk.

## Data Availability Statement

The raw data supporting the conclusions of this article will be made available by the authors, without undue reservation.

## Ethics Statement

Written informed consent was obtained from the individual(s), and minor(s)' legal guardian/next of kin, for the publication of any potentially identifiable images or data included in this article.

## Author Contributions

YZho is the project leader and contributed to all aspects of this work. QP and NZ are joint first authors, who contributed equally to data assembly, analysis, and drafted the manuscript of the present paper. All authors contributed intellectually to this manuscript and have approved this final version.

## Conflict of Interest

The authors declare that the research was conducted in the absence of any commercial or financial relationships that could be construed as a potential conflict of interest.
