# Supplementary material for: Geographical Variation of COPD Mortality and Related Risk Factors in Jiading District, Shanghai
Source: Front Public Health. 2021 Feb 3;9:627312. doi: 10.3389/fpubh.2021.627312 (PMC7888271; doi:10.3389/fpubh.2021.627312)
Supplement: Supplementary file 1 [file Table_1.docx]

Table S1. The association of COPD mortality and risk factors from multivariate logistical regressions by selection of control and season.

| Variables | Multivariate analysis^1^ | |  | Multivariate analysis^2^ | |  | Multivariate analysis^3^ | |  | Multivariate analysis^4^ | |
| --- | --- | --- | --- | --- | --- | --- | --- | --- | --- | --- | --- |
|  | _a_OR(95%CI) | P |  | _a_OR(95%CI) | P |  | _a_OR(95%CI) | P |  | _a_OR(95%CI) | P |
| Education |  |  |  |  |  |  |  |  |  |  |  |
| Senior high school or above |  |  |  |  |  |  |  |  |  |  |  |
| junior middle school |  |  |  |  |  |  |  |  |  |  |  |
| Primary School or below |  |  |  |  |  |  |  |  |  |  |  |
| Marriage |  |  |  |  |  |  |  |  |  |  |  |
| Others |  |  |  |  |  |  |  |  |  | 1 |  |
| Married |  |  |  |  |  |  |  |  |  | 0.587(0.443-0.777) | <0.001^*^ |
| Occupation |  |  |  |  |  |  |  |  |  |  |  |
| Others |  |  |  |  |  |  |  |  |  |  |  |
| Industry- related |  |  |  |  |  |  |  |  |  |  |  |
| Agriculture-related |  |  |  |  |  |  |  |  |  |  |  |
| Extremely low temperature (℃) |  |  |  |  |  |  |  |  |  |  |  |
| >20 | 1 |  |  | 1 |  |  | 1 |  |  | 1 |  |
| 15-20 | 1.429(0.900-2.271) | 0.131 |  | 1.066(0.699-1.624) | 0.768 |  | 0.868(0.529-1.424) | 0.575 |  | 1.036(0.637-1.687) | 0.886 |
| 5-15 | 1.149(0.780-1.693) | 0.483 |  | 1.946(1.321-2.867) | 0.001^*^ |  | 1.191(0.846-1.676) | 0.317 |  | 1.463(1.045-2.050) | 0.027^*^ |
| <5 | 1.557(1.106-2.194) | 0.011^*^ |  | 2.126(1.535-2.945) | <0.001^*^ |  | 1.509(1.039-2.190) | 0.031^*^ |  | 2.133(1.456-3.125) | <0.001^*^ |
| Distance to the closest highway(m) |  |  |  |  |  |  |  |  |  |  |  |
| >650 | 1 |  |  | 1 |  |  | 1 |  |  | 1 |  |
| 350-650 | 1.857(1.248-2.765) | 0.002^*^ |  | 1.402(0.964-2.041) | 0.077 |  | 1.382(0.906-2.108) | 0.133 |  | 1.237(0.805-1.903) | 0.332 |
| 200-350 | 1.580(1.006-2.479) | 0.047^*^ |  | 1.756(1.120-2.752) | 0.014^*^ |  | 2.006(1.275-3.157) | 0.003^*^ |  | 2.604(1.606-4.222) | <0.001^*^ |
| <200 | 2.650(1.391-5.048) | 0.003^*^ |  | 1.450(0.803-2.616) | 0.218 |  | 1.167(0.568-2.400) | 0.674 |  | 0.757(0.384-1.493) | 0.422 |
| Distance to the closest major road(m) |  |  |  |  |  |  |  |  |  |  |  |
| >500 |  |  |  |  |  |  |  |  |  |  |  |
| 300-500 |  |  |  |  |  |  |  |  |  |  |  |
| 100-300 |  |  |  |  |  |  |  |  |  |  |  |
| <100 |  |  |  |  |  |  |  |  |  |  |  |
| Distance to the closest hospital(m) |  |  |  |  |  |  |  |  |  |  |  |
| >2000 | 1 |  |  |  |  |  |  |  |  |  |  |
| 1000-2000 | 1.550(1.082-2.221) | 0.017^*^ |  |  |  |  |  |  |  |  |  |
| 500-1000 | 1.275(0.867-1.877) | 0.217 |  |  |  |  |  |  |  |  |  |
| 200-500 | 1.418(0.908-2.213) | 0.124 |  |  |  |  |  |  |  |  |  |
| <200 | 0.465(0.196-1.103) | 0.082 |  |  |  |  |  |  |  |  |  |
| Distance to the closest factory(m) |  |  |  |  |  |  |  |  |  |  |  |
| >500 |  |  |  |  |  |  |  |  |  |  |  |
| 200-500 |  |  |  |  |  |  |  |  |  |  |  |
| 50-200 |  |  |  |  |  |  |  |  |  |  |  |
| <50 |  |  |  |  |  |  |  |  |  |  |  |
| Factory density |  |  |  |  |  |  |  |  |  |  |  |
| Q4 |  |  |  |  |  |  |  |  |  |  |  |
| Q3 |  |  |  |  |  |  |  |  |  |  |  |
| Q2 |  |  |  |  |  |  |  |  |  |  |  |
| Q1 |  |  |  |  |  |  |  |  |  |  |  |
| Population density |  |  |  |  |  |  |  |  |  |  |  |
| Q4 | 1 |  |  |  |  |  |  |  |  |  |  |
| Q3 | 0.570(0.382-0.852) | 0.006^*^ |  |  |  |  |  |  |  |  |  |
| Q2 | 0.749(0.435-1.289) | 0.296 |  |  |  |  |  |  |  |  |  |
| Q1 | 1.316(0.779-2.223) | 0.305 |  |  |  |  |  |  |  |  |  |
| GDP |  |  |  |  |  |  |  |  |  |  |  |
| Q4 | 1 |  |  | 1 |  |  | 1 |  |  | 1 |  |
| Q3 | 0.952(0.638-1.420) | 0.808 |  | 0.784(0.519-1.183) | 0.246 |  | 1.204(0.789-1.836) | 0.389 |  | 1.330(0.877-2.018) | 0.180 |
| Q2 | 1.002(0.618-1.625) | 0.994 |  | 0.847(0.527-1.362) | 0.494 |  | 1.267(0.755-2.124) | 0.370 |  | 1.370(0.914-2.054) | 0.128 |
| Q1 | 2.015(1.156-3.512) | 0.013^*^ |  | 1.276(1.012-1.423) | 0.038^*^ |  | 2.670(1.486-4.799) | 0.001^*^ |  | 2.011(1.314-3.080) | 0.001^*^ |

^1^Multivariate analysis used data in Autumn and winter ( from September to February), and controls were deaths due to cardiovascular disease.

^2^Multivariate analysis used data in Autumn and winter ( from September to February), and controls were deaths due to tumor.

^3^Multivariate analysis used data in Spring and Summer( from March to August), and controls were deaths due to cardiovascular disease.

^4^Multivariate analysis used data in Spring and Summer( from March to August), and controls were deaths due to tumor.

Temperature represented the average extreme low temperature in the month prior to death.

Q1–Q4 represent the interquartile value of risk factors.

OR, Odds ratio; aOR, adjusted odds ratio; CI, confidence interval.

*P < 0.05.
